# Echocardiographic assessment of left ventricular function in mules under general anesthesia induced with a high-dose xylazine-based protocol

**DOI:** 10.14202/vetworld.2025.1936-1943

**Published:** 2025-07-17

**Authors:** Pannawat Puangsubsin, Ashannut Isawirodom, Porrakote Rungsri, Nuttapon Satumay, Aree Laikul, Worakij Cherdchutham

**Affiliations:** 1 Department of Large Animal and Wildlife Clinical Sciences, Faculty of Veterinary Medicine, Kasetsart University, Kamphaeng Saen Campus, Nakhon Pathom, Thailand; 2 Department of Companion Animal and Wildlife Clinic, Chiang Mai University, Chiang Mai, Thailand; 3 Veterinarian of the Royal Stable Unit, The Royal Thai Army, Bangkok, Thailand

**Keywords:** anesthesia, echocardiography, ejection fraction, fractional shortening, mule, ventricular function, xylazine

## Abstract

**Background and Aim:**

Echocardiographic assessment in equines is typically performed on standing animals; however, no studies have evaluated left ventricular function in anesthetized mules using high-dose xylazine. Given the unique pharmacokinetics in mules and their higher anesthetic requirements, this study aimed to assess the effects of acepromazine-xylazine-diazepam-ketamine anesthesia, using the upper limit xylazine dose (1.6 mg/kg), on the left ventricular size and function in mules.

**Materials and Methods:**

Six healthy adult mules (18.83 ± 0.75 years; 263.83 ± 39.34 kg) were evaluated using standard two-dimensional and M-mode transthoracic echocardiography. Measurements were obtained before sedation (standing) and 13-min post-anesthetic induction (dorsal recumbency). Each mule received an intravenous injection of acepromazine (0.04 mg/kg), xylazine (1.6 mg/kg), diazepam (0.1 mg/kg), and ketamine (2.2 mg/kg). Key echocardiographic parameters included interventricular septum thickness (interventricular septum in diastole and interventricular septum in systole), left ventricular internal diameters (left ventricular internal diameter in diastole and left ventricular internal diameter in systole [LVIDs]), posterior wall thickness (left ventricular posterior wall in diastole and left ventricular posterior wall in systole), ejection fraction (EF), and fractional shortening (FS). Statistical comparisons were made using paired t-tests and Wilcoxon signed-rank tests (p < 0.05).

**Results:**

Heart rate, EF, and FS significantly decreased post-anesthesia (p < 0.01), indicating reduced systolic function. Specifically, LVIDs increased from 4.60 ± 0.65 cm to 6.26 ± 0.48 cm (p < 0.01), while no significant changes were observed in diastolic parameters or respiratory rate. Anesthetic induction was smooth and graded as good to excellent in all cases.

**Conclusion:**

High-dose xylazine significantly suppressed systolic cardiac function in anesthetized mules without causing arrhythmias or bradyarrhythmia. The combination protocol was effective and provided safe anesthesia induction, with echocardiography proving feasible under dorsal recumbency. These findings support the cautious use of upper-limit xylazine dosing in mules and suggest echocardiographic monitoring as a valuable tool during anesthesia.

## INTRODUCTION

The mule, a hybrid offspring of a male donkey and a female horse, possesses distinct physiological and pharmacokinetic characteristics that necessitate adju-stments in anesthetic protocols typically designed for horses [1]. Standard sedative and anesthetic regimens for mules often incorporate alpha-2 adrenergic agonists, such as xylazine, detomidine, or romifidine, combined with the dissociative agent ketamine for short-duration procedures [1]. Field anesthesia protocols commonly employ a combination of xylazine, diazepam, and keta-mine [2]. However, significant interspecies differences, including the faster drug metabolism observed in mules compared to horses [3], highlight the need for species-specific anesthetic strategies. Moreover, mules typically exhibit muted stress responses and physical rea-ctivity [1], further influencing anesthetic planning and dosage requirements. Acepromazine, a phenothiazine tranquilizer, is often administered as a premedicant to enhance the sedative effects of xylazine [1]. In mules, xylazine-induced sedation occurs within 15 min, but it is generally less profound and shorter in duration than that observed in horses [3]. Consequently, increased xylazine dosing – up to 1.6 mg/kg – is often recommended to achieve satisfactory sedation in mules [1]. Despite its efficacy, xylazine is known to exert dose-dependent cardiovascular effects, including bradyarrhythmia and decreased cardiac output [4]. Diazepam, widely used in equine anesthesia for its muscle-relaxant and anxi-olytic properties, enhances the smoothness of anest-hesia when paired with ketamine [5, 6]. Its action on gamma-aminobutyric acid (GABA) receptors promotes muscle relaxation and reduces rigidity associated with induction. In combination protocols, diazepam helps mitigate the risks of bradycardia and hypotension [1, 6]. Although ketamine provides stable anesthesia with minimal cardiovascular depression in horses, mules often experience shorter and less satisfactory anesthetic durations, necessitating careful dosing or rep- eated administration [1]. Echocardiography is a non-invasive and clinically valuable tool for assessing equine cardiac function. It offers real-time measurements of indices such as ejection fraction (EF) and fractional shortening (FS), which, respectively, indicate left ventricular systolic function and contractility [7]. Performing echo-cardiography in non-sedated animals maintains phy- siological hemodynamics and anatomical orientation [8]. Prior echocardiographic studies in mules have been limited to standing animals, such as those with cardiac remodeling due to hyperthyroidism [9]. However, a study by Brown *et al*. [9] did not assess cardiac function under general anesthesia. Although previous research by Dar and Gupta [2] has explored anesthesia and monitoring in mules, none have incorporated echocardiographic evaluations under anesthesia.

Although the pharmacological differences between horses and mules are increasingly acknowledged in veterinary literature, there remains a significant gap in the understanding of cardiovascular responses to general anesthesia in mules, particularly under the influence of high-dose alpha-2 adrenergic agonists. Previous studies have focused on sedative and anesthetic protocols in standing mules or have extrapolated equine data to mule populations without accounting for species-specific differences in drug metabolism, cardiovascular physiology, and anesthetic tolerance. Notably, echocar-diographic evaluations in mules have been limited to baseline cardiac function in unsedated or hyperthyroid animals, with no published data examining real-time changes in left ventricular size and function under anesthesia. Furthermore, while xylazine is widely used in field settings, the hemodynamic consequences of administering its upper recommended dose (1.6 mg/kg) in mules have not been documented using objective cardiac imaging modalities. This lack of data restricts the development of optimized anesthetic protocols and limits the safe application of cardiovascular monitoring techniques in clinical and field conditions.

The present study aims to bridge this knowledge gap by evaluating the effects of acepromazine-xyla-zine-diazepam-ketamine anesthesia, specifically using the upper-limit xylazine dose, on echocardiographic parameters in mules. By employing standardized two-dimensional and M-mode echocardiography, the study investigates changes in key indices of left ventricular function, including interventricular septal thickness, left ventricular internal dimensions, EF, and FS, before and during anesthesia. The research seeks to determine whether this anesthetic combination induces significant alterations in systolic and diastolic function and whether these changes remain within clinically acceptable limits for safe anesthetic use. The findings are expected to inform evidence-based anesthesia practices for mules and contribute to the development of species-specific cardiovascular monitoring protocols during general anesthesia.

## MATERIALS AND METHODS

### Ethical approval

The study was approved by the Institutional Animal Care and Use Ethics Committee of Kasetsart University, Bangkok, Thailand (Approval ID: ACKU67-VET-002).

### Study period and location

This study was conducted from January 2024 to March 2024 at the Equine Medical and Rehabilitation Service Center, Bangkok, Thailand.

### Study design

Six clinically healthy adult mules (four males and two females), aged 18.83 ± 0.75 years and weighing 263.83 ± 39.34 kg, were enrolled in this pilot study. All mules were crossbred from Australian donkeys and Thoroughbred mares and had been previously trained for military use before being retired at the Veterinary Remount Department of the Royal Thai Army in Chiang Mai. Complete blood counts and serum biochemistry profiles were evaluated to exclude any underlying pathology before inclusion. A within-subject, repeated-measures design was used to compare left ventricular size and functional parameters before and after anes-thesia in the same animals.

### Animal housing and management

All animals were transported to the Equine Medical and Rehabilitation Service Center in Bangkok, Thailand, and acclimated for at least 4 days before examination. Each mule was individually housed in a 16 m^2^ well-ventilated box stall without bedding, in preparation for anesthesia. The feeding protocol consisted of 1.3% of the body weight in roughage, divided into four meals daily, with *ad libitum* access to water. Concentrated feed was limited to 1 kg/mule/day.

### Pre-anesthetic echocardiographic examination

**Figure 1:**
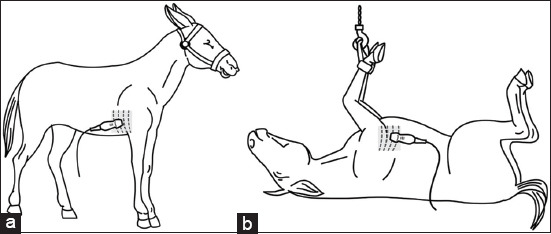
Illustration of echocardiographic probe positioning in two mule positions. The gray-shaded area with dashed lines marks the third to fifth intercostal spaces, where the transducer was positioned to evaluate the left ventricle. (a) Right parasternal window in a standing mule. (b) Right parasternal window in a dorsally recumbent anesthetized mule.

**Figure 2:**
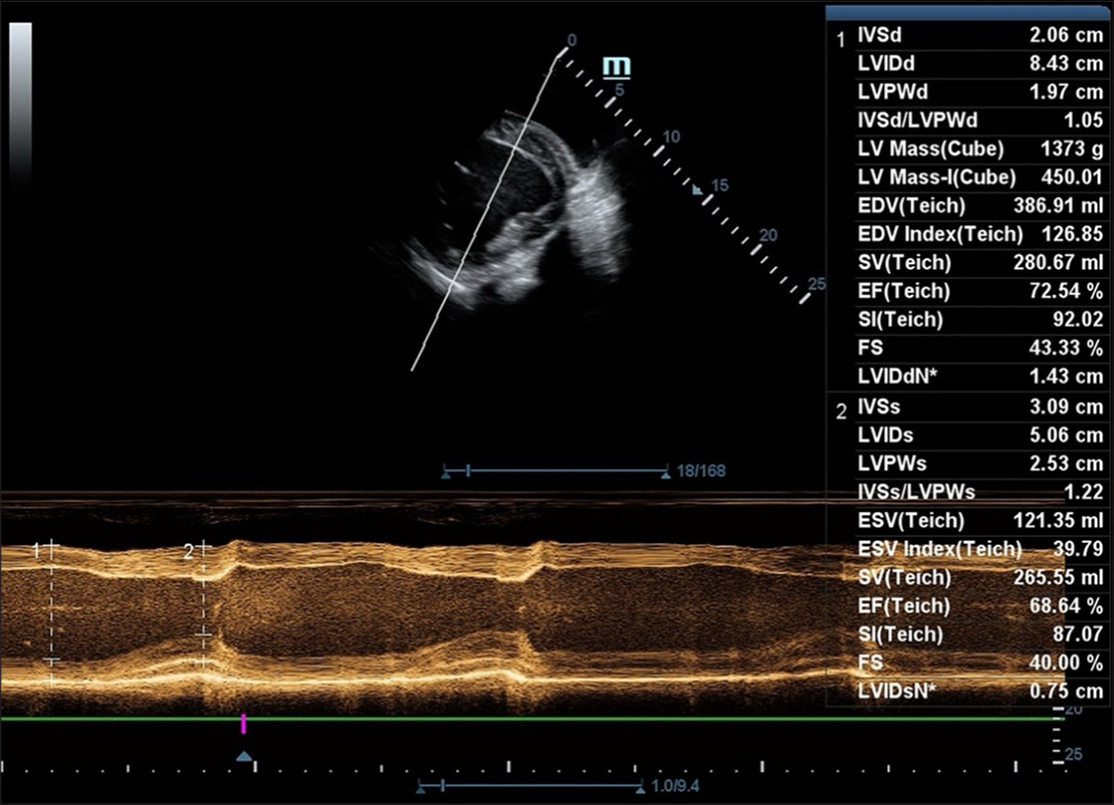
Images of the right parasternal short axis view at the papillary muscle level during diastole and during systole obtained from the pre-anesthesia period in non-sedated standing mules. At an imaging depth of 25 cm, the transducer is pointed next to the olecranon, rotating the transducer 90° clockwise from a four-chamber view and tilted dorsally to optimize visualization of the left ventricle.

Baseline echocardiographic evaluations were performed on unsedated, standing mules using stan-dardized imaging protocols adapted from equine stu-dies. The right third to fifth intercostal spaces were clipped to facilitate optimal probe contact (Figure 1a). A 3.4-MHz phased-array transducer (Vetus 8; Mindray Bio-medical Electronics Co., Ltd., China) was used to acquire images in both two-dimensional and M-mode. Long-axis four-chamber views and short-axis views at the papillary muscle level were obtained as described previously by Reef *et al*. [10], Tucker *et al*. [11], and Decloedt *et al*. [12]. The M-mode cursor was aligned to bisect the left ventricular chamber symmetrically (Figure 2). Images were stored digitally and analyzed offline using the ultrasound machine’s built-in calipers. All measurements were performed in triplicate by a single experienced operator to minimize variability.

### Anesthesia protocol and induction

Food was withheld for 10–12 h before anesthesia, although water remained available. Baseline vital para-meters, including heart rate (HR) and respiratory rate (RR), were recorded. A jugular venous catheter was placed for drug administration. Each mule received intravenous acepromazine maleate (0.04 mg/kg; Comb-istress, Kela N.V., Belgium), followed 15 min later by xylazine hydrochloride (1.6 mg/kg; X-LAZINE, L.B.S. Laboratory Ltd., Thailand). Anesthesia was induced using diazepam (0.1 mg/kg IV; Ropam, L.B.S. Laboratory Ltd.,) and ketamine (2.2 mg/kg IV; Ketamine-hameln, Hameln Pharma GmbH, Germany). Induction quality was video recorded and scored using standardized criteria [13]. Animals were hoisted and placed in dorsal recumbency on a padded table with the head and neck extended to ensure airway patency.

### Echocardiographic assessment during anesthesia

Echocardiographic examinations were repeated once animals reached stage 3, plane 2 of anesthesia; confirmed by absent palpebral reflex; and preserved corneal reflex and ventromedial eye rotation. The transducer was repositioned (Figure 1b) to obtain right parasternal long-axis four-chamber views and short-axis views at the papillary muscle level, with the probe rotated 90° clockwise and tilted dorsally [12]. All asse-ssments were performed by the same operator to main-tain consistency.

### Echocardiographic parameters and calculations

The following M-mode parameters were mea- sured: Interventricular septum in diastole (IVSd) and systole (IVSs), left ventricular internal diameter in diast-ole (LVIDd) and systole (LVIDs), and left ventricular posterior wall thickness in diastole (LVPWd) and systole (LVPWs) (Table 1) [10, 11]. Functional indices were calc-ulated as follows:

**Table 1:** Abbreviations and descriptions of selected echocardiographic parameters [10, 11].

Abbreviation	Parameter	Description
Diastolic measurement (cm)		
IVSd	The interventricular septum during diastole	Interventricular septum thickness during diastole
LVIDd	Left ventricular internal diameter during diastole	Internal diameter of the LV during diastole.
LVPWd	Left ventricular posterior wall in diastole	Thickness of the posterior wall of the LV during diastole.
Systolic measurement (cm)		
IVSs	The interventricular septum during systole	Interventricular septum thickness during systole
LVIDs	Left ventricular internal diameter during systole	Internal diameter of the LV during systole.
LVPWs	Left ventricular posterior wall in systole	Thickness of the posterior wall of the LV during systole.
Volume parameters (mL)		
LVEDV	Left ventricular end-diastolic volume	Volume of blood in the LV at the end of diastole, before contraction
LVESV	Left ventricular end-systolic volume	Volume of blood remaining in the LV at the end of systole after contraction
Functional parameters (%)		
EF	Ejection fraction	Percentage of blood ejected from the LV
FS	Fractional shortening	Percentage reduction in left ventricular diameter from diastole to systole

LV=Left ventricle

FS = ([LVIDd–LVIDs] ÷ LVIDd) × 100

EF = ([LVEDV–LVESV] ÷ LVEDV) × 100

**Figure 3:**
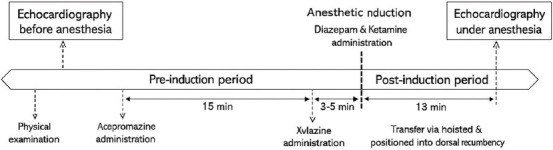
The schematic of anesthetic protocol and echocardiographic timeline for all mules.

Left ventricular volumes (LVEDV and LVESV) were derived using the Teichholz formula programmed in the ultrasound software. The echocardiographic timeline and anesthetic protocol are summarized in Figure 3.

### Statistical analysis

Normality of data for HR, RR, and echocardio-graphic parameters was assessed using the Shapiro–Wilk test. Paired t-tests were used for normally distributed data, while the Wilcoxon signed-rank test was applied to non-parametric variables. Parametric data are presented as mean ± standard deviation and non-parametric data as median ± interquartile range. Statistical analyses were performed using the Statistical Package for the Social Sciences version 30.0 (IBM Corp., Armonk, NY, USA), with significance defined as p < 0.05.

## RESULTS

### Timing and quality of anesthetic induction

Echocardiographic assessments were performed 13 min after induction, following the full transfer and positioning of mules in dorsal recumbency. This timing coincided with the anticipated peak effect of anesthesia. Anesthetic induction was rapid, occurring within appro-ximately 1 min, and was consistently graded as good to excellent across all subjects.

### Cardiopulmonary variables

HR showed a statistically significant decrease from 50.33 ± 6.47 bpm to 40.00 ± 7.90 bpm during anesthesia (p < 0.01), as shown in Table 2. No arrhythmias or conduction abnormalities were detected during the monitoring period. RR did not differ significantly between the pre-anesthesia and anesthesia phases.

**Table 2:** Comparison of HR, RR, and selected echocardiographic parameters between pre-anesthesia and at 13 min after anesthesia periods in dorsal recumbency position in mules (n = 6).

Parameter	Pre-anesthesia			Anesthesia			p-value
	
	Mean ± SD	Min	Max	Mean ± SD	Min	Max	
HR (bpm)	50.33 ± 6.47	42.00	60.00	40.00 ± 7.90	30.00	54.00	<0.01*
RR (bpm)	26.5 ± 3.99	20.00	32.00	22.33 ± 4.63	18.00	28.00	0.17
IVSd (cm)	2.12 ± 0.35	1.69	2.69	1.97 ± 0.34	1.64	2.55	0.13
LVIDd (cm)	8.00 ± 0.99	6.55	9.43	8.30 ± 0.84	7.52	9.51	0.28
LVPWd (cm)	2.25 ± 0.61	1.94	2.76	2.31 ± 0.86	1.69	4.04	0.75^a^
IVSs (cm)	3.13 ± 0.52	2.39	3.91	3.05 ± 0.73	2.44	4.42	0.36
LVIDs (cm)	4.60 ± 0.65	3.58	5.39	6.26 ± 0.48	5.59	6.81	<0.01*
LVPWs (cm)	3.58 ± 0.90	2.72	3.88	3.16 ± 0.86	2.53	4.87	0.35 ^a^
EF (%)	71.11 ± 4.01	66.22	75.96	46.18 ± 4.58	39.68	52.27	<0.01*
FS (%)	42.30 ± 3.06	38.88	46.29	24.28 ± 2.99	20.16	28.35	<0.01*

a Data are not normally distributed, so Wilcoxon signed-rank test p-value used and reported as median ± interquartile range.

* Statistically significant (p < 0.05) mean values from the paired t-test. HR=Heart rate: RR=Respiratory rate, IVSd=Interventricular septum in diastole, IVSs=Interventricular septum in systole, LVIDd=Left ventricular internal diameter in diastole, LVIDs=Left ventricular internal diameter in systole, and LVPWd=Left ventricular posterior wall thickness in diastole, LVPWs=Left ventricular posterior wall thickness in systole, EF=Ejection fraction, FS=Fractional shortening

### Changes in left ventricular function

**Figure 4:**
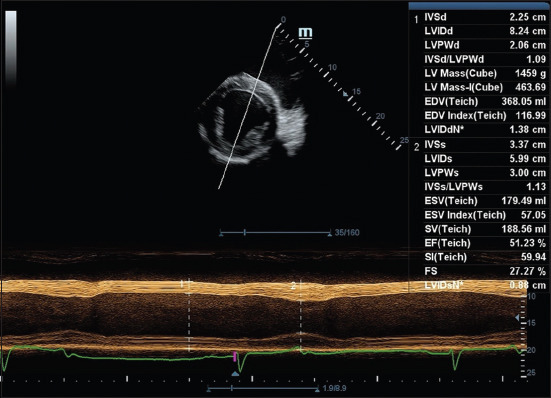
Images of the right parasternal short axis view at the papillary muscle level during diastole and during systole obtained from the post-induction period at 13 min of anesthesia in the dorsal recumbent mules. At an imaging depth of 25 cm, the transducer is pointed next to the olecranon with an upside-down transducer placement position and directions, rotating the transducer 90° clockwise from a four-chamber view and tilted ventrally to optimize visualization of the left ventricle.

Echocardiographic measurements from the right parasternal short-axis LV echocardiography during anesthesia are shown in Figure 4. Both EF and FS declined significantly during anes-thesia, indicating a reduction in systolic performance. The only systolic structural parameter to show a signif-icant change was the LVIDs, which increased from 4.60 ± 0.65 cm to 6.26 ± 0.48 cm (p < 0.01), further supporting evidence of diminished contractility.

### Stability of other ventricular measurements

No significant changes were noted in other systolic parameters, including the IVSs and the LVPWs. In addition, diastolic measurements – IVSd, LVIDd, and LVPWd – remained statistically unaltered between pre-and post-anesthesia evaluations.

## DISCUSSION

### Baseline imaging and anesthetic strategy

Baseline echocardiographic imaging was condu-cted in unsedated mules to maintain physiological card-iac function and anatomical orientation. This approach enabled accurate comparison of cardiac parameters before and after anesthesia. The anesthetic protocol, comprising acepromazine, xylazine, diazepam, and keta-mine, reflected standard clinical practice in equine and mule anesthesia [1, 2] and resulted in uniformly smooth inductions. All drug doses adhered to recommended guidelines for mules [1].

### Pharmacological effects of acepromazine

While acepromazine alone does not provide deep sedation, it enhances anesthetic consistency by dampening catecholaminergic responses [14]. It also reduces myocardial sensitivity to catecholamines, potentially lowering the risk of fatal arrhythmias and anesthetic-related mortality [15]. Prior studies indicate that acepromazine minimally affects HR and blood pressure and does not significantly alter cardiac output [16, 17]. Although vasodilation and blood pressure reduction have been reported, echocardio-graphic parameters, including IVSs, LVIDs, LVPWs, IVSd, LVIDd, and LVPWd remain largely unchanged following acepromazine administration at 0.1 mg/kg [16–18].

### Cardiovascular effects of xylazine

Xylazine, an alpha-2 adrenergic agonist, suppresses norepinephrine release, reducing sympathetic tone and increasing parasympathetic activity [2, 4]. In this study, the observed significant reduction in HR aligns with findings in both horses [19] and mules [2] anesthetized with xylazine–ketamine protocols. Despite a high dose (1.6 mg/kg), bradyarrhythmias were not detected, suggesting species-specific cardiovascular tolerance. Notably, only LVIDs increased significantly, while EF and FS decreased, indicating compromised systolic function. These effects are consistent with known cardiovascular depression induced by xylazine [4, 20].

Diastolic measurements, including IVSd and LVIDd, remained relatively stable, suggesting that preload was not substantially affected, likely due to a xylazine-induced decrease in HR [16]. High inter-individual varia-bility may explain the absence of statistical significance in the changes of LVPWd and LVPWs [16].

### Synergistic effects and literature correlation

The combination of acepromazine with xylazine has been shown to significantly reduce FS within 15 min of administration [21], supporting the depressant effects observed in this study. In the study by Ibrahim *et al*. [22] donkeys receiving epidural xylazine at lower doses (0.2 mg/kg) also reported transient reductions in FS and EF. Similarly, intravenous doses of xylazine ≥1 mg/kg in horses have demonstrated reductions in contractility and cardiac output, with increased risk of arrhythmia and bradycardia [4, 23, 24]. In contrast, our study observed no bradyarrhythmia even at a dose of 1.6 mg/kg, reinforcing the possible interspecies cardiovascular resilience in mules.

### Diazepam and ketamine modulation

Diazepam, a gamma-aminobutyric acid agonist, contributes to muscle relaxation and exerts minimal cardiovascular depression [5]. When combined with keta-mine, it may help stabilize hemodynamic responses [25]. Although ketamine typically increases HR by enhancing sympathetic tone [25], its effect in this study was likely suppressed by xylazine’s dominant parasympathetic influence [26]. As previously reported by Sage *et al*. [27], HR decreases in animals anesthetized with xylazine–ketamine combinations, consistent with our findings.

### Comparison to published echocardiographic ranges

The echocardiographic values recorded were consistent with published reference ranges for healthy equids. Measurements of IVSd, IVSs, LVIDd, and LVPWd were within normal reference intervals reported in horses, donkeys, and mules [10, 11, 28, 29]. Similarly, EF and FS values, ranging from 55% to 70% and 30% to 45%, respectively, aligned with those seen in both standing and anesthetized equines [8].

### Impact of dorsal recumbency and anatomical variability

Dorsal recumbency can significantly influence echocardiographic measurements due to shifts in organ positioning and respiratory mechanics. Gravitational pressure on the caudal vena cava may restrict venous return, altering cardiac output and systolic function [30]. Lower body weights in mules, compared to horses, may reduce the risk of muscle damage (e.g., rhabdomy-opathy) associated with recumbency [31]. Nonetheless, this position presents technical challenges during echocardiography due to chest movement and changes in acoustic windows, which can potentially cause variability in measured cardiac indices [8].

## CONCLUSION

This study provides the first echocardiographic evaluation of left ventricular size and function in mules under general anesthesia induced with a high-dose xylazine-based protocol. Key findings included a significant reduction in HR, EF, and FS, along with a marked increase in LVIDs, indicating depressed sys-tolic function. Diastolic indices remained statistically unchanged, suggesting minimal impact on ventricular filling.

The anesthetic regimen of acepromazine (0.04 mg/kg), xylazine (1.6 mg/kg), diazepam (0.1 mg/kg), and ketamine (2.2 mg/kg) was effective for safe induction, with all mules achieving smooth anesthesia without bradyarrhythmia or conduction abnormalities. These findings support the use of ech-ocardiography as a valuable monitoring tool in field or clinical settings, especially when using high-dose alpha-2 agonists in stoic animal such as mules.

The study employed a standardized within-subject design, used clinically relevant drug combinations, and ensured consistency through single-operator echoc-ardiography and repeated measures. It also adhered to established equine imaging protocols, enhancing the reliability of interspecies comparisons.

The small sample size (n = 6) and the use of aged, retired mules may limit the generalizability of the res-ults. In addition, echocardiographic assessments were conducted at a single post-induction time point, which restricted the evaluation of temporal changes across anesthetic depth.

Larger-scale studies with diverse age groups and multiple time-point assessments are warranted to further delineate the hemodynamic effects of varying xylazine doses. Comparative evaluations of alternative sedatives and anesthetic combinations in mules could refine guidelines for anesthetic safety and efficacy.

Despite inherent physiological differences bet-ween mules and horses, the high-dose xylazine-based protocol demonstrated acceptable cardiovascular safety and predictable anesthetic quality. Echocardiography emerges as a feasible and informative modality for real-time intra-anesthetic monitoring in mules, offering an evidence-based approach to optimizing equine hybrid anesthesia protocols.

## AUTHORS’ CONTRIBUTIONS

PP: Designed the study, analyzed the data, and writing-reviewing. AI: Echocardiography. PR, NS, and AL: Study coordination, data recording. WC: Con-ceptualized and supervised the study and revised the manuscript. All authors have read and approved the final manuscript.
